# RETRACTION: “3D Culture System for Liver Tissue Mimicking Hepatic Plates for Improvement of Human Hepatocyte (C3A) Function and Polarity”

**DOI:** 10.1155/bmri/9763128

**Published:** 2026-04-24

**Authors:** BioMed Research International

RETRACTION: Z. Jia, Y. Cheng, X. Jiang, C. Zhang, G. Wang, J. Xu, Y. Li, Q. Peng, and Y. Gao, “3D Culture System for Liver Tissue Mimicking Hepatic Plates for Improvement of Human Hepatocyte (C3A) Function and Polarity,” *BioMed Research International* 2020, no. 1 (2020): 6354183, https://doi.org/10.1155/2020/6354183.

The above article, published online on 04 March 2020 in Wiley Online Library (wileyonlinelibrary.com), has been retracted by agreement between; the journal Editor, Vitale Miceli; and John Wiley & Sons Ltd. The retraction has been agreed after the following figure concerns were noted:

− In Figure 3A, the image for the panel, ‘Group S, Day 1’ was duplicated in panel ‘Group S, Day 3’;
− In Figure 6C, the bottom left image of "Group M, Day 7" and the top left image of "Group H, Day 13" share unexpected similarities.

The authors requested a correction to Figure 3A and initially disagreed that there were unexpected similarities in Figure 6C. However, the editor believes the images in Figure 6C were generated from the same tissue section of a single sample and do not represent independent samples taken at two different time points. When questioned further, the authors stated that the similarities in Figure 6C were present due to an error during the figure preparation.

Further investigation also noted unexpected similarities between Figure 1E and Figure 3A. As such, the editor does not have confidence in the results, and so this article is retracted.

The authors disagree with this decision.

